# Addressing Different Needs: The Challenges Faced by India as the Largest Vaccine Manufacturer While Conducting the World’s Biggest COVID-19 Vaccination Campaign

**DOI:** 10.3390/epidemiologia2030032

**Published:** 2021-09-17

**Authors:** Cinja Nadana Koller, Cléo Josephine Schwerzmann, Alexia Suzanne Aimée Lang, Eleni Alexiou, Jaya Krishnakumar

**Affiliations:** 1 Global Studies Institute, University of Geneva, 1205 Geneva, Switzerland; cleo.schwerzmann@etu.unige.ch (C.J.S.); alexia.lang@etu.unige.ch (A.S.A.L.); eleni.alexiou@etu.unige.ch (E.A.); 2 Institute of Economics and Econometrics, Geneva School of Economics and Management, University of Geneva, 1211 Geneva, Switzerland; Jaya.Krishnakumar@unige.ch

**Keywords:** India, COVID-19, vaccination strategy, vaccine manufacturing, vaccine exports, vaccine diplomacy

## Abstract

The COVID-19 pandemic has highlighted some of the challenges that countries face when balancing domestic and global necessities, for example with regard to vaccine needs, production and distribution. As India hosts one of the world’s largest vaccine manufacturing industries and has one of the most extensive vaccination strategies, the country is particularly exposed to these challenges. This has become all the more obvious as the country experienced a second pandemic wave in the first half of 2021, which has led to a total ban on exports of COVID-19 vaccines. An analysis of the national vaccination strategy and the domestic vaccine industry through review of peer-reviewed literature, grey literature, and news reports showed the fragile balance between domestic and international needs. A numerical comparison of India’s domestic COVID-19 vaccine needs, export agreements, and production capacities was conducted. It was found that at current production rates as of April 2021, meeting all of the needs and complying with all of the agreements would be impossible. Scale-ups in production, as promised by the industry, however, will enable the achievement of all targets in the long term.

## 1. Introduction

With over 1.3 billion inhabitants, India is the second biggest nation in the world and the biggest among low- and middle-income countries in terms of population [1,2]. India has experienced substantial economic growth over the past 20 years, including strong growth of the pharmaceutical industry [2,3]. According to the Indian Brand Equity Foundation (IBEF), India has the third biggest pharmaceutical industry by volume produced and produces more than half of the world’s vaccines [3]. Nevertheless, the country’s spending on public health is still relatively low, leading to challenges in tackling public health issues and achieving universal health coverage [4].

In December 2019, a cluster of pneumonia of unknown cause was found in China; this turned out to be a Coronavirus Disease (COVID-19) caused by a contagious new virus (SARS-CoV-2). Following its rapid spread, on 30 January 2020, the outbreak was declared a public health emergency of international concern by the WHO’s Director-General [5]. On the same day, the first case of COVID-19 was confirmed in India [6]. In the following months, the Indian government imposed restrictions on public life and international travel, for example by suspending visas and promoting and implementing social distancing measures [7,8]. On 24 March 2020, the Government of India issued a strict lockdown to contain the spread of the disease through uniform measures [9]. Nevertheless, until April 2021, the country would register over 14 million cases and an estimated 175,000 deaths [10].

Since the beginning of the SARS-CoV-2 outbreak, various vaccines have been developed around the world and in India, including a first locally developed vaccine called Covaxin. Covaxin was developed by the Indian company Bharat Biotech (Hyderabad, India). More vaccine candidates are still in the pipeline in other companies. India being home to a large pharmaceutical industry and the world’s biggest vaccine manufacturer, the Serum Institute of India (SII), (Pune, India) [11], found itself in an interesting but challenging position regarding the manufacturing and distribution of COVID-19 vaccines domestically as well as internationally.

The following article will look at the domestic needs of India as well as its international positioning as a vaccine manufacturer, aiming to assess the challenges and opportunities the country is facing. In the first part, the article describes the COVID-19 situation during the second wave in India, followed by a discussion of the world’s largest Covid-19 vaccination campaign launched by the Indian government. Then, the various COVID-19 vaccine developments in India as well as the production capacities of Oxford AstraZeneca’s Covishield and Bharat Biotech’s Covaxin in India are assessed. The article further highlights international export agreements of COVID-19 vaccines, with a focus on the COVAX agreement and the initiative for an intellectual property rights waiver. Three future scenarios, based on the collected data, are analyzed and graphically represented. The discussion of the compatibility of India’s goals as a COVID-19 vaccine producer, exporter and national government delivering vaccines to its population concludes that not all goals are simultaneously achievable.

India was hit hard during the second wave of the Covid-19 pandemic. As one of the largest Covid-19 vaccine manufacturers and exporters, India is contributing to ending the pandemic around the world. The country is helping other low- and middle-income countries through COVAX exports and commercially exporting vaccines while also conducting the world’s largest vaccination campaign. These goals are ambitious, and stakeholders’ interests seem to be competing. This led to the investigation of the compatibility of India’s vaccine-related manufacturing and exporting goals [12].

### Current Situation in India

India is currently facing a second wave of COVID-19 cases, and the country is listed second on the list for the number of deaths by country, counting 211,853 deaths in total by the end of April 2021. In addition, on 30 April 2021, India reported 401,993 confirmed cases in one day, which is the highest single-day spike so far [12]. The driving factor of this outcome is considered to be the new homegrown variant, B.1.617, which includes a mutation that makes the virus extremely contagious [13].

According to Pandey and Nazmi [14], the devastating spread of the virus affected big cities first, but rapidly reached smaller towns and rural areas as well. It is reported that hospitals have reached full capacity, and several have faced acute shortage of oxygen and critical drugs. Lockdown has been re-introduced in several States as well. On the other hand, launching the largest vaccination campaign, India started vaccinating its population on 16 January 2021, and declared that more than 150 million doses had been administered by the beginning of April [10,15].

While dealing with the world’s biggest COVID-19 crisis, many States also reported a shortage of vaccines. On 29 April 2021, the Health Minister of Maharashtra State, Mr. Rajesh Tope, mentioned that, even though the State is able to immunize 0.8 million people per day, their stock has only been sufficient for around 0.7 million, and expressed his concerns about the upcoming third phase of the vaccination campaign (people aged 18–44 years old) that starts on 1 May 2021 [16]. Thus, it appears that the country that hosts the world’s largest vaccine manufacturer is currently facing problems on stocking and distributing vaccines domestically, which puts obstacles in the way of the vaccination rollout. Additionally, the U.S. restricted exports of raw materials for vaccine production, preserving them for its own companies and causing delays in the vaccine production at the SII [17].

On March 24, the Government of India temporarily halted all exports of the Covishield vaccine manufactured by the SII until the country’s situation stabilize and domestic needs are covered [18]. This was a decision that immediately affected the distribution of vaccines through the COVAX facility, and the WHO announced that there would be potential delays in securing and distributing Covishield doses that had been agreed upon in advance to the 82 beneficiary countries [19]. At the same time, India proceeded to grant emergency approval to the Sputnik V vaccine developed by Moscow’s Gamaleya Institute (Moscow, Russia), which made agreements for its production with six domestic vaccine manufacturers [11].

Towards the end of April, India received international aid for medical supplies such as ventilators and oxygen containers from the UK, the European Union, Denmark, the UAE, Saudi Arabia, Bhutan, and Australia. Moreover, the President of the USA engaged in providing rapid support in supplies. The U.S. ban on exports of vaccine raw materials mentioned earlier was also loosened for India [20].

## 2. Materials and Methods

This case report will review the existing literature, data, and news reports on COVID-19 vaccines in India. The article will compare the country’s domestic needs, its capacity to produce and its obligations to export produced vaccines. Through this comparison, the analysis aims to answer the following research question: when positioning itself as one of the main global manufacturers of the COVID-19 vaccine while also executing the world’s largest scale COVID-19 vaccination program, is India capable to produce, administer and export COVID-19 vaccines at the same time? For the literature research, scientific articles as well as articles from major local and international newspapers were used. Only articles dealing with the vaccination production, schedule, export or the second wave were considered. Articles focused on the first wave were excluded. The Indian Government’s website, the ministries’ websites such as the Ministry of Health and Family welfare, as well as UN agencies’ websites, were used as the main sources for figures and recent events. Since the situation is still ongoing, the data is constantly evolving. For this paper, real-time data was considered until the end of April 2021. Numbers from the aforementioned sources were used to calculate possible future scenarios, which are also represented graphically. The paper mainly focuses on the vaccines that are currently approved for use in India and are internationally traded. All amounts in Indian Rupees were converted into US $ using the exchange rate of 27 May 2021 ($1 = Rs 72.71/Re 1 = $0.01375) [21].

## 3. Results

As mentioned above, India is a country with a population of over 1.3 billion people, that has faced a catastrophic second wave of COVID-19. In addition, the country hosts the biggest vaccine manufacturer in charge of most vaccine exports from India, while also supplying COVID-19 vaccines for its large domestic needs. In order to assess India’s ability to succeed with its plans, this study analyses three main factors: the national vaccination strategy, the vaccine production and the international contracts and commitments made regarding vaccine exports.

### 3.1. Vaccination Strategy

In a press release, India announced that the vaccination campaign had reached the benchmark of 6 million vaccinated people only 24 days after starting the nationwide COVID vaccination program [22]. Besides, the press release from the Ministry of Health and Family Welfare emphasized that India had been the fastest country in the world to achieve this number. By August 2021, at least 300 million of the 1.38 billion people living in India are expected to be vaccinated [23]. To roll out the world’s largest COVID-19 vaccination campaign, the Indian government introduced the National Expert Group on Vaccine Administration for COVID-19 (NEGVAC) and published a detailed document named COVID-19 Vaccines Operational Guidelines [24]. Already having experience with large immunization programs, India ensures in their COVID-19 strategy that the different governance levels—national, State and district—are working together [24]. Not only collaboration but also digital solutions are used to make the campaign as efficient as possible. A digital platform named Co-WIN (COVID-19 Vaccine Intelligence Network) has been developed for Indian citizens to register for their immunization shots [24]. In addition, responsible persons in the vaccination program can create an account or session sites and facilities can be entered into the system [24]. Moreover, the platform allows real time data tracking, including immunization tracking and lets beneficiaries control the progress of the vaccination program [24].

In order to register for a vaccination slot, any individual has to provide basic personal data such as current address, date of birth or co-morbidities, as well as a picture for identification verification before receiving the shot. At the session sites, vaccination officers verify the pre-registered people and update their vaccination status after they have been immunized, using the Co-WIN application [24].

**Figure 1:**
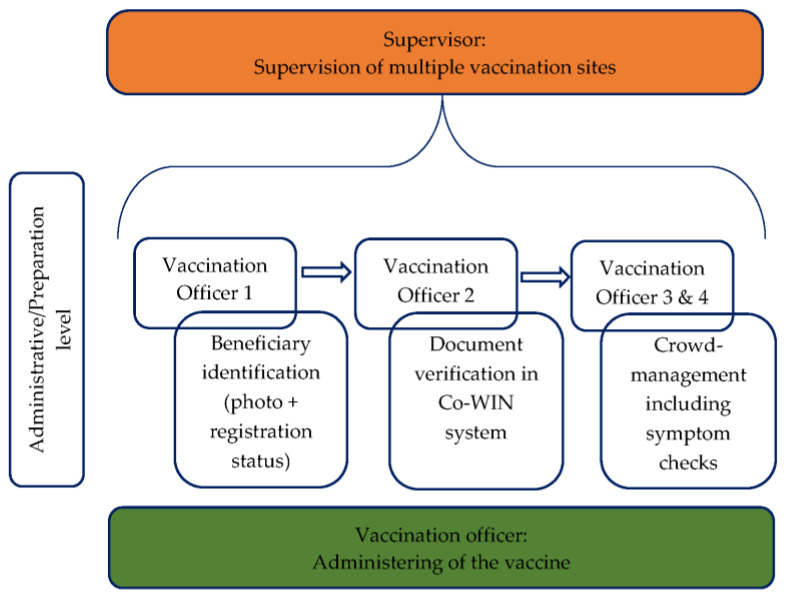
Roles of vaccination team. Own illustration, data compiled from Ministry of Health and Family Welfare Government of India [24].

Before India started to actively vaccinate, any involved staff engaged in large training programs. These trainings were mostly organized virtually at the State and district level. Furthermore, self-learning modules were provided for frontline COVID-19 workers. Different personnel such as healthcare staff, program managers, cold chain handlers, or vaccinator officers were included in training adapted to their specific tasks. Generally, roles in the vaccination process were distributed as in the following five categories shown in Figure 1.

Only after a district unit completed the full training were they allowed to start the COVID-19 vaccination [24]. Throughout the vaccination process, supervisors who are responsible for the smooth running and support of districts progressing slowly accompany the vaccination sites and teams [24].

Although the WHO recommends a prioritization of vaccinating people older than the age of 60 in order to achieve the highest reduction in COVID-19 mortality [25], NEGVAC decided to start with the vaccination of healthcare personnel [24]. However, through mathematical modeling, it was found that the best strategy to reduce COVID-19 mortality in India would be to vaccinate the over 65 years first, as recommended by WHO [25,26]. Nevertheless, India’s weak healthcare system and shortage of healthcare workers justify the chosen strategy by NEGVAC because this strategy will protect the fragile healthcare system during the pandemic [26]. This strategy is in line with recommendations for countries who engage in COVAX [25]. As India does so, the Ministry of Health and Family Welfare of India emphasized “global equitable access to a vaccine, particularly protecting healthcare workers and those most-at-risk is the only way to mitigate the public health and economic impact of the pandemic and is the current priority” [24]. Therefore, in a first phase, vaccines will be administered to healthcare workers, frontline workers, and the high-risk population. These three groups account for around 30 million people. The second group of frontline workers includes, for example, staff from the Ministry of Defense, the police, or municipal workers. People older than 50 years or people under 50 years old but with comorbidities such as hypertension or lung disease fall under the category of high-risk population. Moreover, Indian States may be able to prioritize vaccine distribution; in a case where a specific region shows high numbers of COVID-19 infections, States are allowed to favor this region, but only within the three identified priority groups [24].

Between 2021 and 2022, $10.17 billion of the Union budgets were allocated to the Ministry of Health and Family Welfare, the majority of which is expected to be used reinforce the COVID-19 vaccination program [27]. The Government of India decided to provide the COVID-19 vaccines to immunize healthcare workers and frontline personnel for free [23]. Vaccination will be free of charge for the population if the vaccination center is State-run. If the vaccination is administered in private health institutions, part of the costs must be borne by the patients and the institution [28]. One dose of Covishield costs the Indian government around $2.75, while the Covaxin costs around $2.83 per dose [23]. With the aim to vaccinate 300 million people with two doses by August 2021 and the current production capacity of 87.5% Covishield and 12.5% Covaxin, the cost for the government would be approximately $1,656,000,000 if the doses were provided for free to the broad population (Equations (1)–(4)):

(1) 300,000,000×2=600,000,000=87.5 % Covishield+12.5 % Covaxin

(2) 600,000,000×0.875=525,000,000

(3) 600,000,000×0.125=75,000,000

(4) ($2.75×525,000,000)+($2.83×75,000,000)=$1,656,000,000

The Ministry of Health and Family Welfare of India calculates the required doses with the following formula [24]:

(5) X=(Y×Z÷V)×2×WMF

where *X* represents the COVID-19 vaccine required for one month, *Y* is the total population to be covered in the relevant catchment area (State/district/block/sector), *Z* is the proportion of population to be covered in this catchment area, *V* is the number of months of the campaign, and *WMF* equals the Wastage Multiplication Factor for the COVID-19 vaccine, assuming an allowable programmatic wastage of 10%:

(6) WMF=100÷(100−wastage)=100÷(100−10)=100÷90=1.1

Additionally, various international organizations provide technical and monitoring services to support the health system in India, especially during the implementation of the vaccination campaign. This includes the WHO, for example, who assist with the training of staff and UNICEF who help with capacity building in the cold-chain management [24].

As of 30 April 2021 no information was available on how the government is planning to proceed with the vaccination strategy after the 300 million mark is reached.

### 3.2. Vaccine Manufacturing

Prior to the COVID-19 pandemic, India was producing around 60% of the world’s vaccines. Therefore, India positions itself as one of the leaders in COVID-19 vaccine manufacturing worldwide [29]. The country’s industry has been contracted to manufacture several COVID-19 vaccines and has also developed their own. They are large contributors to vaccine distribution at the international level. Indeed, India exported about 64 million doses of COVID-19 vaccines to more than 70 countries between mid-January and March [30,31]. In this section, the aim is to assess the role of the government in the vaccine development, production, and distribution. A particular focus will be put on the interconnection and decision-making power between the government and private laboratories during the manufacturing and distribution of the vaccines.

Table 1 reports the five different vaccines that can be manufactured in India, including Covishield, Covaxin and Sputnik V which were approved for use within the country as of April 2021 [11].

The three vaccines Covishield (AstraZeneca, Cambridge, UK), COVID-19 Vaccine Janssen (Johnson & Johnson, New Brunswick, NJ, USA), and Sputnik-V (Gamaleya National Center, Moscow, Russia) from international patent holding companies are manufactured and distributed by companies in India through voluntary licenses [33]. Under a voluntary license, the patent holder gives the authorization to a third party to manufacture and distribute the patented vaccines after agreeing on mutually beneficial conditions [34].

Approval of vaccines is the duty of the Central Drugs Standard Control Organization, the Directorate General of Health Services, and the Ministry of Health and Family Welfare, which are the main governmental departments involved in drafting regulatory guidelines for development of vaccines. Vaccine developers are guided by the official “guidelines for development of vaccines with special consideration for COVID-19 vaccine”, which aim to ensure that vaccines are well-characterized, constantly manufactured and appropriately stored. They also provide specific instructions to establish safety and protective immunity in clinical trials as well as a defined post-marketing surveillance strategy which includes assessment of Adverse Events Following Immunization (AEFI) and Adverse Events of Special Interest (AESI). To be approved, the vaccines must meet the following endpoints: the prevention of clinically apparent infections that fit the primary case definition based on clinical and laboratory criteria; special consideration for Efficacy of COVID-19 Vaccines and Statistical Considerations; safety considerations are assessed prior to the approval of the vaccine. Once the vaccines are approved, post-marketing clinical evaluation is performed to monitor vaccine safety in routine use [35].

The Government of India, to confront the pandemic, needed to undertake a vaccine deployment strategy. To do so, the first step is to estimate the country’s needs for vaccines. As calculated in Section 3.1, to vaccinate 300 million people with two doses each until August 2021 the cost would be approximately $1.656 billion dollars. It is also important to consider that the production of COVID-19 vaccines must not be conducted to the detriment of other existing domestically produced vaccines. The country’s future needs may also be influenced by the population’s demand for vaccines: vaccine hesitancy, for example, can slow down a vaccination campaign, as could be observed in India at the beginning of the vaccine rollout [36]. Due to the fact that demand has since increased substantially, this paper will not further discuss the challenges regarding the demand [37].

To reach the goal of herd immunity at the country level, the constraint on India’s vaccine manufacturers had to be alleviated through the development of strategies aiming at the optimization of the production. Through the “Mission COVID Suraksha’’, launched by the Government of India, the development of vaccine candidates was accelerated, ensuring the optimization of the process until licensure and introduction to the market. The mission follows and facilitates the end-to-end focus from pre-clinical to clinical development and manufacturing and regulatory facilitation [38].

As part of Mission Covid Suraksha, the Government of India allocated $123,750,000 in Research & Development grants for COVID-19 vaccine development on 12 November 2020. In April 2021, Dr. Renu Swarup, Secretary of the Department of Biotechnology at the Ministry of Science and Technology, reported that $68,750,000 had been allocated to five vaccine candidates and numerous research facilities. Further grants for increase in production capacity were being considered, however, the sums required by the industry exceed any grants offered so far [39].

Private party agreements led to the collaboration of the SII and AstraZeneca to produce Covishield and Dr. Reddy’s Laboratories’ partnering with Russia’s Sputnik V vaccine. Additionally, direct partnerships between vaccine developer firms and the government enabled the development and production of Indian-made vaccines through indigenous vaccine research. Collaboration between developers, clinical trial units and vaccine manufacturers can facilitate this [40].

The government plays a central role in the COVID-19 vaccine development and regulation. Therefore, the government implemented a four-pronged strategy on a State-private sector partnership model for speeding up vaccine development and production [41].

The four pillars of this strategy are:

1. Financial support for vaccine producers;
2. Institutional help for vaccine producers throughout the drug development cycle;
3. Optimized pathway to vaccine approval;
4. Boost of production through ‘out-of-the-box’ solutions [41].

To meet the terms of the Mission Covid Suraksha, various means of financial support were allocated to the manufacturing and developing companies, essentially supporting the increase in production capacity. This support included grants by the Government of India for vaccine manufacturers producing indigenous vaccines [42]. Under this initiative, Bharat Biotech, for example, received $8,937,500 million specifically to enhance its production capacity [27]. Moreover, Bharat Biotech and the SII received advance payments from the Government [27]. These payments amounted to $206.25 and $412.5 million, respectively [27].

From January to April 2021, India was using two Indian manufactured vaccines: Oxford-AstraZeneca’s Covishield, which currently produces up to 840 million of vaccines per year, and Bharat Biotech’s COVAXIN, which produces 120 million doses per year at present [37,42]. Since mid-April, emergency use authorization was provided to Russia’s Sputnik V vaccine through agreements with local pharmaceutical companies, as India is a main production hub for this vaccine (Gland Pharma, Hyderabad, India; Hetero Biopharma, Hyderabad, India; Panacea Biotec, Lalru, India; Stelis Biopharma, Bengaluru, India; Virchow Biotech, Hyderabad, India). The production goal for this vaccine would be up to 850 million doses per year [43].

**Figure 2:**
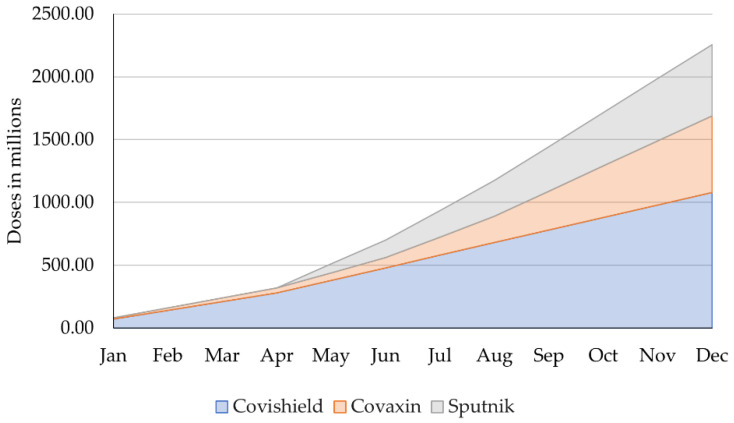
Cumulative production capacity by vaccine type (incl. scale-up). Own illustration, data compiled from Ahmed, Ministry of Science & Technology, and Press Trust of India [37,42,43].

The funds described above would allow for an increase in the monthly capacity of the SII from 70 million doses currently to 100 million doses by the end of May 2021 and would increase the annual production of Bharat Biotech to around 700 million doses [42]. Figure 2 describes the cumulative production capacities including the planned increases in production described above.

To speed up the vaccination program, the government is in the process of authorizing other vaccines already in use in other countries such as the vaccines by Pfizer (New York, NY, USA), Moderna (Cambridge, MA, USA) or Johnson & Johnson (New Brunswick, NJ, USA) [30]. This is a necessary response to the current crisis in which the cases number are dramatically rising concurrently to an increasing shortage of vaccines. The vaccine production is overstressed, and available funds are not enough to increase vaccine production capacity [39]. However, as of now, it is not known whether these new authorizations will be granted. Therefore, the following sections do not include possibility of other vaccines and will only be based on the increased production capacities of the already approved vaccines at the time of writing.

Along with this increase in production, the Government of India aims to start the Liberalised and Accelerated Phase 3 Strategy of COVID-19 Vaccination on 1 May 2021. According to this strategy, the manufacturers would provide 50% of their supply to the Government and the remaining 50% would enter the open market, allowing State governments or private entities (e.g., private hospitals) to procure vaccines [44]. Price transparency on the supply allocated to the open market is required to be announced by the producers prior to 1 May [27].

### 3.3. Vaccine Exports: International Contracts and Relations

As stated above, five different vaccines can be manufactured in India, three of which have been approved for emergency use within the country as of April 2021: Covishield, Covaxin and Sputnik V [11].

Indian vaccine manufacturers have signed various international contracts with different partners. However, even though there are many vaccines and vaccine candidates available, as of April 2021, only two vaccines (Covishield and Covaxin) are actively being exported. As of 28 April 2021, 66.37 million doses have been exported from India through three different pathways: COVAX, commercial agreements and the Vaccine Maitri [31].

The first stakeholder receiving vaccines from Indian manufacturers is COVAX. The COVAX initiative is part of the Access to COVID-19 Tools Accelerator (ACT Accelerator), a collaboration framework of various private and public actors, including the WHO and GAVI, with the goal of accelerating development, production and equitable access to tests, treatments, and vaccines. Its strategy consists of four pillars, one of which is COVAX [45].

COVAX is the pillar supporting COVID-19 vaccine research, development, and manufacturing as well as equitable distribution. COVAX will allow participating countries to access vaccines even if they do not have bilateral deals with manufacturers or lack the financial means to procure vaccines. The initiative’s goal is to globally distribute 2 billion doses, which would allow for the protection of some of the most vulnerable and exposed groups, such as healthcare personnel [46].

The initiative has concluded contracts with the SII, which has pledged to contribute at least 200 million doses of its Covishield vaccine to COVAX in the first half of 2021 [47]. So far, COVAX has only received Covishield vaccines from the SII, however, once approved, the Novavax vaccine is also expected to be delivered [48,49]. At the end of March 2021, 19.86 million doses of Covishield had been delivered through COVAX to some of the 64 recipient countries [31,50].

India holds a special position in this context because, while the country’s industry is delivering vaccines to the COVAX initiative, India is also one of the biggest beneficiaries of it. India is expected to receive around 20% of the doses provided through COVAX [51]. This means that not all of the vaccine doses exported to COVAX are actually being exported, rather they stay in the country. India is set to receive almost 50 million doses from COVAX, of which the country has already received 10 million doses [51]. These deliveries are part of the vaccines dispatched by Indian manufacturers [50].

Numerous manufacturers have made further bilateral commercial deals with foreign countries. However, there is little publicly available data on the exact nature of these deals. The Ministry of External Affairs states that during the first quarter of 2021, 35.79 million doses were exported through commercial agreements. The exported doses were mainly Covishield vaccines; only 760,000 doses of Covaxin have been exported through this pathway so far [31].

As described earlier in this article, equitable access to vaccines is imperative for the fight of the COVID-19 pandemic. However, many high-income countries have been securing doses through commercial agreements with manufacturers. By December 2020, several of them had contracts for vaccines covering more than their own populations [52]. Low-and middle-income countries, on the other hand, are falling short in the competitive market; this market also influenced COVAX in their efforts to secure vaccines, while only trying to reach their goal of enough vaccines for 20% of the people in beneficiary countries [53].

In response to that, the Indian government launched a vaccine diplomacy program called the “Vaccine Maitri” [54]. The program delivers millions of vaccine doses to other low-and middle-income countries around the world free of cost, based on the idea that no Indians are safe until everyone is safe [54]. Recipient countries include Nepal, Bhutan, Morocco and Afghanistan [31]. As of 28 April 2021, over 10 million doses have been dispatched to five continents [31].

**Figure 3:**
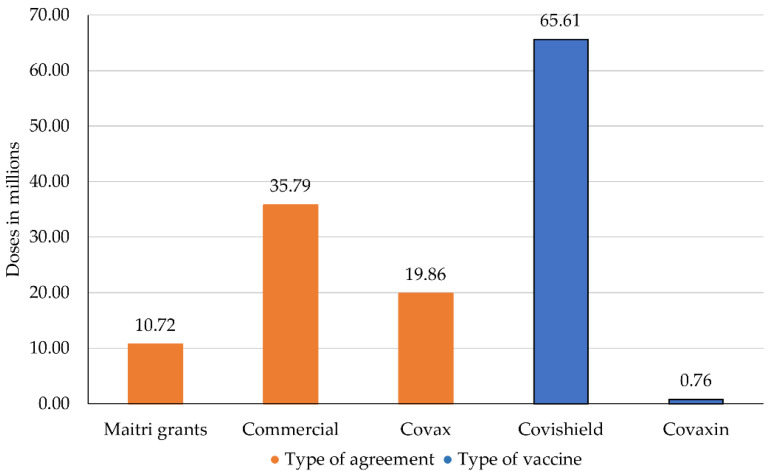
Exported COVID-19 vaccines by type of agreement and type of vaccine. Own illustration, data compiled from Ministry of Science & Technology, and Press Trust of India [42,43].

Figure 3 provides an overview over the exported vaccine doses described in the previous paragraphs.

Under the circumstances of the second wave and the beginning of the largest vaccination roll-out, India halted the exports of the Covishield vaccine [55]. This decision was made domestically by the Indian government and the SII in order to prioritize the domestic need for vaccinations [56]. Therefore, vaccine deliveries were delayed, which affected mostly low-and middle-income countries that were to receive vaccines through COVAX [57]. Despite the delays, GAVI supported India’s decision and, along with the WHO, they called on the EU countries and other high-income countries to show solidarity and start sharing vaccines [58]. To sum up, the absence of legally binding mechanisms to engage the market actors to perform beyond their benefit, and the weakness in coordinating the management of resources seemed to raise concerns regarding the equitable access of high-income countries and low-and middle-income countries to the COVID-19 vaccines [59]. In addition, this issue highlighted the gaps and weaknesses of the global governance system, especially when it comes to vaccine distribution. The issue further showed the necessity of improvement on the road to universal health coverage [60].

In other international efforts, India, along with South Africa, is trying to make access to vaccines and medicines possible for all countries by demanding an intellectual property rights waiver. The two countries called on all WTO members to repeal certain parts of the Trade-Related Aspects of Intellectual Property Rights (TRIPS) Agreement in order to strengthen the fight against COVID-19 worldwide in solidarity. The proposal is now supported by many WTO members and has been addressed to the TRIPS Council [61]. On 30 April 2021, the TRIPS council decided to continue discussing the proposal, but its introduction might take some time [61,62]. The IP waiver is intended to enable the production of COVID-19 vaccines and medicines in more middle-income countries. For India, this would allow for more technology transfers, easier access to production materials, and an increase in production capacities [62]. Indeed, as mentioned above, India has the capacity to produce 60% of the world’s vaccines but only a limited number of local companies are currently tech holders and under voluntary licenses. However, inducing a diversification of manufacturers could scale-up the vaccine production [63].

Recently, an important milestone was reached in the waiver process through the support of the USA and other G7 members. Although the waiver is gaining in popularity, drug producers expressed doubts that the lifting of patents would not lead to the desired goal, since vaccine production requires the highest expertise in various fields and would therefore not eliminate the vaccine shortage. For the waiver to enter into force, all 164 members of the WTO must give their consent [62].

## 4. Discussion

By the end of 2020, most countries had already passed through a first wave of COVID-19, and since then, attention has concentrated on vaccine manufacturing and distribution. As described above, India, where five vaccines are being produced, launched the world’s largest vaccination strategy that started rolling out in January 2021 and aimed to administer 600 million doses by August 2021. In order to secure vaccines for its domestic needs, the Indian government provided funding and agreements for the SII and other pharmaceutical companies while also securing international contracts for vaccine exports through the COVAX initiative, commercial agreements, and the Vaccine Maitri program. Moreover, India also benefited in vaccine doses from the COVAX program. Although the country’s vaccination strategy has been very promising, the second wave that struck in March was a deadly hit for India, who concurrently started facing a shortage of vaccines. Under these circumstances, India has been challenged to fulfill the agreements arranged on vaccine exports, while also covering its domestic needs. The Indian government’s reaction was to enable a faster approval of vaccines already used in other countries, increase the production capacity for domestic needs and temporarily halt the exports of the Covishield vaccine, causing delays in the distribution of doses through the international agreements and the COVAX initiative. Taking a prospective view, this section examines the possible scenarios for the balance between production capacity, domestic needs, and export agreements.

Initially, India’s monthly production capacity amounted to 80 million doses of Covishield and Covaxin combined. Thanks to support from the government and international demand, manufacturers have promised the scale-up of their production. As a result, in May, Covishield’s monthly dose production is expected to increase from 70 million to 100 million doses. Gradually, Covaxin’s monthly production is said to increase as well, and in May, the Sputnik V vaccine would join the Indian vaccine market, providing 70.83 million additional doses monthly on average. Producing at this increased pace, 1.08 billion doses of Covishield, 610 million doses of Covaxin and 566.66 million doses of Sputnik V vaccines would be produced until the end of 2021, adding up to a total of 2256.66 billion doses.

According to the national strategy, India’s domestic vaccine needs amount to 600 million doses until August. 152 million doses were administered until April [64]. If the vaccination speed remains similar to April’s for the period from May to August (86.88 million/month), 499.52 million doses would be administered by August:

(7) 152,000,000+86,880,000×4=499,520,000

In this case, the national vaccination target would not be achieved.

In order to calculate the average number of monthly doses that would have to be administered to reach the target of 600 million doses by August, the remaining doses to be administered after April were calculated as shown in Equation (8) and divided by the four months that are left until August (9):

(8) 600,000,000−152,000,000=448,000,000

(9) 448,000,000÷4=112,000,000

Thus, India would have to administer 112 million doses per month to achieve its aim by August 2021.

By the end of April, the export agreements had led to a total of 66.37 million doses deployed from India. A total of 10.72 million vaccines were exported through the Vaccine Maitri program, 35.79 million doses through commercial agreements and 19.86 million doses through COVAX initiative [31]. However, 200 million doses were promised to COVAX until the end of July. In order to achieve this goal, India would have to export 180.14 million doses until July. Regarding the commercial agreements, at this time, no data is available on promised deliveries and, therefore, this aspect is not considered in our calculations. However, this may have some influence on the number of doses exported until the summer.

The following sections will consider three potential outcomes about the vaccination target and the export agreements depending on whether the vaccine production in India will actually be scaled-up after April 2021 and assuming that the country is increasing its vaccination speed to 112 million doses per month Equations (8) and (9) in order to achieve its target.

### 4.1. Scenario 1: Production Capacity Increased as Promised

**Figure 4:**
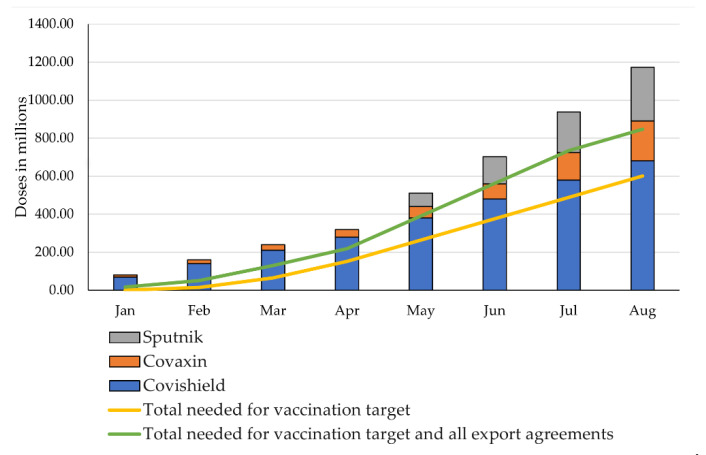
Production capacities (incl. scale-up) vs. vaccination target and total doses needed for all targets. Own illustration, data compiled from Bagcchi, Ministry of External Affairs, Ahmed, Ministry of Science & Technology, Press Trust of India, Serum Institute of India, and Our World in Data [23,31,37,42,43,47,64].

Assuming that the promised increase in the production capacity takes place, 1.173 billion doses will be produced by August 2021. To reach the country’s vaccination target of 600 million doses, 448 million doses remain to be administered, which would be covered by the increased production. (Figure 4).

In this case, production would exceed the domestic needs. In Figure 4, export agreements are also considered. The domestic needs, along with the pending 180.14 million doses for the COVAX facility, amount to 628.14 million doses needed between May and August (Figure 4). The total production to cover the national vaccination target, the COVAX agreement, and the already deployed deliveries through Vaccine Maitri and commercial agreements would have been 846.52 million:

(10) 600,000,000+200,000,000+10,720,000+35,790,000=846,520,000.

Therefore, if the increase in production capacity and the vaccination campaign are executed as promised, there would be no conflict of interest and all parties would receive the necessary doses to reach all targets.

### 4.2. Scenario 2: The Production Remains as It Was until April 2021

**Figure 5:**
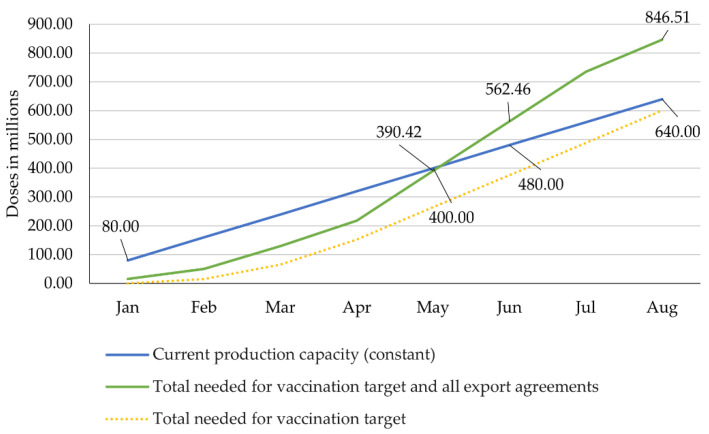
Constant production capacity vs. fulfilment of vaccination target and all export agreements. Own illustration, data compiled from Bagcchi, Ministry of External Affairs, Ahmed, Ministry of Science & Technology, Press Trust of India, Serum Institute of India, and Our World in Data [23,31,37,42,43,47,64].

**Figure 6:**
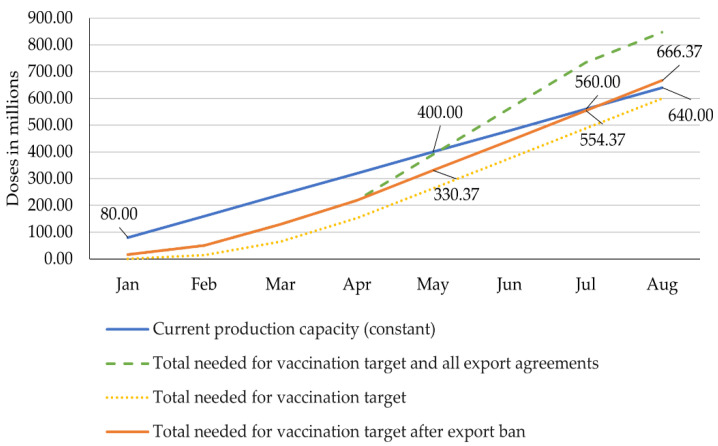
Constant production capacity vs. vaccine needs after export ban in April. Own illustration, data compiled from Bagcchi, Ministry of External Affairs, Ahmed, Ministry of Science & Technology, Press Trust of India, Serum Institute of India, and Our World in Data [23,31,37,42,43,47,64].

The scenarios 2.1 and 2.2 assume the production to remain constant at 80 million doses per month. In this case, 640 million doses would be produced by August (Figure 5 and Figure 6).

#### 4.2.1. Scenario 2.1: Constant Production Capacity vs. Fulfilment of Vaccination Target and All Export Agreements

As shown above, the manufacturers would have to produce 628.14 million doses between May and August to fulfil the COVAX agreement and to allow the country to achieve its vaccination target until the summer. Under the conditions that the production remains constant, if the national vaccination target were to be achieved, and all export agreements were to be met, the country would run out of vaccine doses between May and June: production will have reached 480 million doses by June, but the national vaccination target and the export agreements would require around 562.46 million doses to be met (Figure 5).

In this case, a conflict of interest would be unavoidable and at least some parties would not receive the doses they need. As can be seen in Figure 5, considering only India’s domestic needs, supply would suffice until August to reach the vaccination target.

#### 4.2.2. Scenario 2.2: Constant Production Capacity vs. Vaccine Needs after Export Ban in April

Assuming that the export ban introduced at the end of April 2021 remains in place until August, the total production would have to be 666.37 million doses, which includes the national vaccination target of 600 million and the 66.37 million doses already exported until April. Assuming that the production capacity remains constant, the country will still run out of doses by August. Production will have reached 640 million doses whereas 666.37 million doses will be required to achieve the national target, as displayed in Figure 6.

It is this scenario which, to some extent, provides the justification for the temporary cessation of vaccine exports since in any scenario without scaled-up production, the country would run out of vaccines at a given point in time. However, an export ban allows the country to delay and decrease the gap between supply and needs. In this scenario, it is highly likely that the vaccine manufacturers will be able to sufficiently scale-up production to at least meet India’s domestic needs until August, since only a comparably small scale-up (26.37 million) is needed to reach the 600 million doses targeted (Figure 6).

### 4.3. Limitations

This study has been conducted between March and May 2021, at a time when India was dealing with the second wave of COVID-19 and the vaccination strategy was still under Phase II. As a consequence, there has been a lack of scientific papers on the rollout of the vaccination campaign as well as the COVID-19 vaccine production and exports. Thus, data was collected mostly from official governmental services, WHO updates as well as Indian press and web sources, which sometimes led to conflicting information from various sources. To address this issue, the authors chose to keep the data from the official governmental sources whenever possible, although there may be some concerns regarding their accuracy.

Additionally, the ongoing second wave evolved so rapidly that the current updates on the vaccination needs and capacity, mortality, confirmed cases rates and related policies were dramatically changing every day. Therefore, to be able to analyze possible outcomes based on consistent data, our study and the data were limited to the situation before 30 April 2021, which meant that we did not go on with a further investigation of the evolution of the vaccination needs and capacity for Phase III of the vaccination drive.

## 5. Conclusions

In conclusion, India may face critical challenges while running its large vaccination strategy and remaining the world’s largest vaccine manufacturer. By April 2021, the second wave brought grievous outcomes to the country. Additionally, the country is facing challenges to balance its domestic needs for vaccines while also exporting millions of vaccine doses to various stakeholders. In order to be able to administer 600 million doses by August 2021 as aimed, an increase in the monthly production capacity as of May 2021 was planned. Nevertheless, the Government of India ordered a temporary ban of vaccine exports.

To analyze whether the country can position itself as a main COVID-19 vaccine manufacturer while also executing its mass vaccination program, three scenarios were examined. Their outcomes depend on whether the production accelerates:

1. if it does, the vaccination target and all the export agreements will be accomplished (Figure 4);
2. if it does not, the country will face severe shortage in June at the latest and will not meet either the internal or the export needs (Figure 5), or;
3. if it does not accelerate, but the ban of exports introduced at the end of April continues, the country will still face a shortage in August, which can be avoided with a small increase in production capacity so that the country achieves its vaccination target (Figure 6).

These scenarios do not take into account the possibility of the approval of other locally produced vaccine candidates or the import of foreign vaccines in the meantime, which could enable India to close the gap and even lift the export ban. This might be an interesting analysis to pursue as more information becomes available. Since this case report is a prospective study conducted within a context that was evolving quickly, future studies may be able to use this review as a base to retrospectively evaluate the decisions made about the production scale-up and the export agreements, their impact on the national vaccination campaign and vice versa. Other aspects that may be interesting to explore, including comparisons of vaccine diplomacy in different countries and regions or the further investigation of the waiver for the TRIPS agreement.

All in all, India must continuously re-assess and adapt to the increasing vaccination needs, in order to successfully carry out the world’s largest vaccination strategy and fulfill all the export agreements. Ideally, this way, all the recipients would receive the vaccine doses they need, because “no one is safe until everyone is safe [65].”

## Figures and Tables

**Table 1:** The five different vaccines manufactured in India. Own illustration, data compiled from the BBC, Ministry of Health and Family Welfare [11,24,32].

Product	Indian Distributer	International Collaborator
Covishield	Serum Insititute of India	Astra Zeneca
Covaxin	Bharat Biotech International Ltd.	
Sputnik V	Dr. Reddy’s Laboratories	Gamaleya National Center
NVX-CoV2373	Serum Institute of India	Novavax
COVID-19 Vaccine Janssen	Biological E	Johnson & Johnson
